# Intra‐ and inter‐tooth variation in strontium isotope ratios from prehistoric seals by laser ablation multi‐collector inductively coupled plasma mass spectrometry

**DOI:** 10.1002/rcm.8158

**Published:** 2018-06-26

**Authors:** A. Glykou, G. Eriksson, J. Storå, M. Schmitt, E. Kooijman, K. Lidén

**Affiliations:** ^1^ Archaeological Research Laboratory Stockholm University SE‐10691 Stockholm; ^2^ Osteoarchaeological Research Laboratory Stockholm University SE‐10691 Stockholm; ^3^ Department of Geosciences, Swedish Museum of Natural History, SE‐10405 Stockholm

## Abstract

**Rationale:**

Strontium isotope ratios (^87^Sr/^86^Sr) in modern‐day marine environments are considered to be homogeneous (~0.7092). However, in the Baltic Sea, the Sr ratios are controlled by mixing seawater and continental drainage from major rivers discharging into the Baltic. This pilot study explores if variations in Sr can be detected in marine mammals from archaeological sites in the Baltic Sea.

**Methods:**

^87^Sr/^86^Sr ratios were measured in tooth enamel from three seal species by laser ablation multi‐collector inductively coupled plasma mass spectrometry (LA‐MC‐ICP‐MS). The method enables micro‐sampling of solid materials. This is the first time that the method has been applied to marine samples from archaeological collections.

**Results:**

The analyses showed inter‐tooth ^87^Sr/^86^Sr variation suggesting that different ratios can be detected in different regions of the Baltic Sea. Furthermore, the intra‐tooth variation suggests possible different geographic origin or seasonal movement of seals within different regions in the Baltic Sea through their lifetime.

**Conclusions:**

The method was successfully applied to archaeological marine samples showing that: (1) the ^87^Sr/^86^Sr ratio in marine environments is not uniform, (2) ^87^Sr/^86^Sr differences might reflect differences in ecology and life history of different seal species, and (3) archaeological mobility studies based on ^87^Sr/^86^Sr ratios in humans should therefore be evaluated together with diet reconstruction.

## 1 INTRODUCTION

To establish migratory patterns in prehistoric seals in the Baltic is important for understanding both prehistoric human and seal behaviour and the interaction between them. In this pilot study we make use of the fact that the present‐day Baltic Sea displays large variations in ^87^Sr/^86^Sr in different parts of the basin. Based on this, and the fact that there are distinct differences in social, foraging and breeding behaviour of the three seal species included in this pilot study, we predict that sequential *in situ* measurement of ^87^Sr/^86^Sr isotope ratios in seal teeth by laser ablation multi‐collector inductively coupled plasma mass spectrometry (LA‐MC‐ICP‐MS) can provide data on life cycle and migratory patterns within and among species.

**Figure 1:**
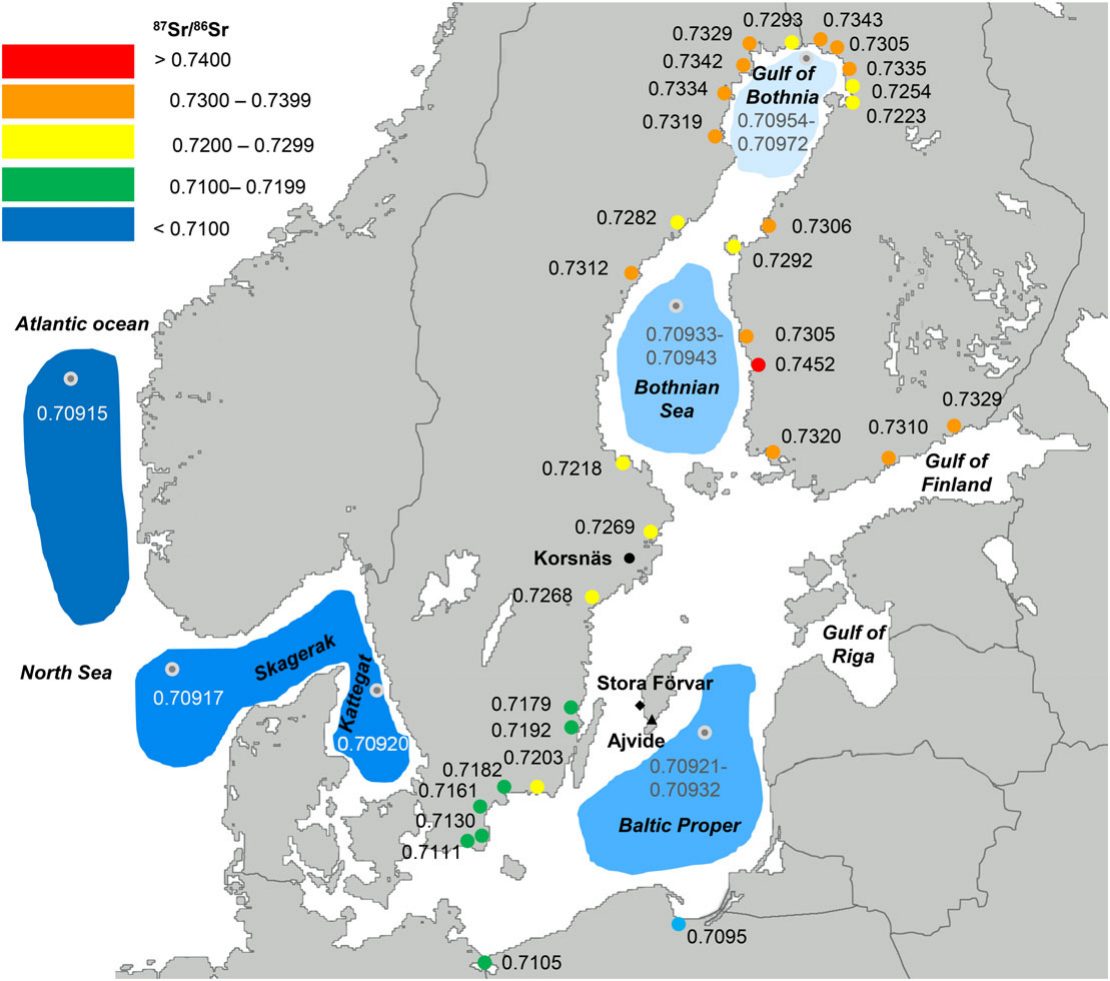
Map with colour‐coded ^87^Sr/^86^Sr values in five intervals. Grey dots indicate the approximate position of water sampling stations after Andersson et al.^4^ The coloured dots indicate the approximate discharge area of the rivers and their ^87^Sr/^86^Sr values after Löfvendahl et al^3^ [Color figure can be viewed at http://wileyonlinelibrary.com]

The ^87^Sr/^86^Sr in oceanic seawater is globally homogeneous within the limits of analytical precision, averaging 0.70918. This is due to a long residence time of Sr in the oceans (5.0 × 10^6^ years) that causes Sr to homogenize isotopically by mixing on a timescale of about 1000 years.^1^ However, in coastal areas, and in smaller basins such as the Baltic Sea, the ^87^Sr/^86^Sr ratios can vary considerably. The present‐day Baltic Sea is a brackish water body, but its complex natural history has involved fluctuations between freshwater and brackish/marine conditions during the Holocene and it is not clear to what extent this has affected the Sr isotope composition. Today, there is a major inflow of freshwater as approximately 50 rivers discharge into the basin.^2^ The rivers drain various sediments ranging from Precambrian in the north to Phanerozoic in the south, thus having different Sr isotope ratios (Figure 1).^3^, ^4^ Accordingly, the rocks in the north have low Sr concentrations and high ^87^Sr/^86^Sr ratios (0.7111–0.7452), whereas in the south they have high Sr concentrations and low ^87^Sr/^86^Sr ratios, closer to that of seawater (0.7095–0.7105).^2^, ^3^ In the water areas where rivers with high Sr concentrations discharge, a bias towards Sr river water concentrations is expected, as deviations from perfect mixing with seawater have been observed.^4^ Despite the high ^87^Sr/^86^Sr ratios in rivers in the north, their relatively low Sr concentrations lead to a lesser influence on ^87^Sr/^86^Sr in the Gulf of Bothnia (0.7095–0.7097) or the Bothnian Sea (0.7093–0.7094), than in the Baltic Proper (0.7092–0.7093), where the rivers have higher Sr concentrations (Figure 1). Consequently, modern mollusk shells collected along the shores reveal high ^87^Sr/^86^Sr in the Bothnian Bay (0.7098–0.7106), Bothnian Sea (0.7094–0.7095), Baltic Proper (0.7093–0.7094), and Kattegat (0.7092).^5^, ^6^ The ^87^Sr/^86^Sr of water in the region of Kattegat is very close to that of seawater, due to water exchange with the North Sea and the Atlantic Ocean.^4^

Strontium has been extensively used in archaeology to elucidate questions of human and animal mobility. Understanding mobility patterns helps us to reconstruct resource acquisition strategies, social networks and interactions between groups, e.g. trading with goods.^7^ Although previous studies of mobility in prehistoric people have assumed that the marine signature is uniform in all environments,^8^ the variation seen in ^87^Sr/^86^Sr in the Baltic suggests that studies of people in coastal areas who feed extensively on marine mammals may be affected by this.

Different seal species have different ecological requirements and display distinctive foraging and migrating patterns. In the present‐day Baltic Sea, there are three species of seal: the grey seal (Halichoerus grypus), the ringed seal (Phoca hispida) and the harbour seal (Phoca vitulina). However, in prehistory there was also the harp seal (Phoca groenlandica). In the present study, teeth from grey seal, ringed seal and harp seal are investigated.

The Baltic Sea population of grey seals is one of three modern grey seal populations that exist independently from each other and have different breeding areas. The other two are the grey seal populations of the western North Atlantic and eastern North Atlantic.^9^ The grey seal population of the Baltic Sea breeds in groups at the margin of the fast ice in the Gulfs of Bothnia, Riga and Finland, but breeding on rocky islets has also been recorded during extremely mild winters,^9^, ^10^ demonstrating highly adaptive behavior. The grey seal is a non‐migratory species, spending most of its time in the proximity of the breeding area but it has been recorded that some, especially subadult, individuals can cover long distances in search of food.^9^, ^11^, ^12^ The Baltic Sea population of grey seals breeds in February–March and lactation lasts approximately 3–4 weeks.^13^

Harp seal, by contrast, is an ice‐dependent and highly migratory species. Today, there are three populations of harp seals with breeding grounds in the White Sea, the Jan Mayen Islands and around Newfoundland. Every year they form large moulting rookeries during spring, and after moulting is completed they start their annual migration northwards to the summer feeding grounds to return to their breeding grounds in the late autumn.^14^ They aggregate in many thousands to give birth on pack ice and lactation lasts 10–12 days.^14^ During the Litorina stage of the Baltic Sea, harp seals were breeding probably in the region between Gotland and the Åland islands, and in the southwestern Baltic Sea.^15^, ^16^, ^17^ It is not yet clear when the breeding population of the Baltic Sea became extinct, but harp seal bones are recorded in the archaeological record until to the Bronze Age, c. 1000–800 cal BC, and they appear sporadically during the Iron Age.^18^, ^19^, ^20^

The ringed seal is a circumpolar species, found along the Arctic coasts, which preferably inhabits inshore waters. The population of the Baltic Sea is distributed in the Bothnia Bay, the Archipelago Sea, and the Gulfs of Riga and Finland. It is solitary and stationary, and maintains breathing holes in fast ice areas during the winter. Between mid‐March and mid‐April pups are born in snow lairs. Lactation lasts up to 2 months.^13^

## 2 EXPERIMENTAL

### 2.1 Sample collection

We sampled six seal teeth in total (five canines and one molar) from grey seal, harp seal and ringed seal (Table 1). Five of the samples are from archaeological collections dating to the Stone Age: one from the site of Korsnäs, one from Ajvide and three samples from Stora Förvar^21^, ^22^ (Figure 1, Table 1). One sample is from a modern grey seal from the Baltic Sea. The selection of the samples was based on the grounds that different species have different ecological requirements and display different foraging and migration patterns. In addition, it enabled testing if regional differences in ^87^Sr/^86^Sr can be detected in seals from different sites.

**Table 1:** Description of the samples

Sample	Site	Species	Tooth	Tooth length (mm)	Pulp cavity	Biological age	Baltic stage	Archaeological period/culture
015	Stora förvar	grey seal	canine	25	open	<3 months	Ancylus/Littorina	Mesolithic
006	Stora förvar	ringed seal	canine	19	closed	>12	Ancylus/Littorina	Mesolithic
013	Stora förvar	grey seal	molar	nd	closed	>12	Ancylus/Littorina	Mesolithic
009	Korsnäs	harp seal	canine	28	closed	>12 months	Littorina	Middle Neolithic/Pitted Ware Culture
029	Ajvide	harp seal	canine	22	open	<6 months	Littorina	Middle Neolithic/Pitted Ware Culture
004	(modern, Baltic Sea)	grey seal	canine	26	open	<3 months	Baltic Sea	(modern)

Measurements on teeth length after Bowen et al^25^ and Hewer et al^26^

### 2.2 Ageing of the seals according to teeth

In most phocid seals the deciduous teeth are shed *in utero*, so that the permanent dentition is already erupted at the time of birth, or erupts shortly after birth.^23^, ^24^

In harp seals, the lower canines erupt a week after birth.^25^ The canines grow rapidly from an average length at birth of 15.3 ± 0.53 mm to an average length of 26.1 ± 0.84 mm at the age of 1 year. The pulp canal has an average diameter of 6.6 ± 0.6 mm at birth and decreases rapidly within the first 12 months as it becomes filled with dentine. Based on the canine length of 22 mm, the seal from Ajvide (sample 029) was estimated to be younger than 6 months, since harp seal canines within 6 months reach 93% of their length at the age of 1 year. However, as the pulp canal measured 6 mm, corresponding to measurements of newborns, the estimated age is probably considerably less than 6 months. The second harp seal canine, from Korsnäs, has a total length of 28 mm and the pulp cavity is closed, suggesting that it belonged to an animal older than 12 months (Table 1).

The length of canines of newborn grey seals is about 16–22 mm. The tooth is not fully erupted until the pups are weaned at approximately 3–4 weeks; the canines have by then grown another 5–7 mm.^26^ The canine length at 6–8 months is 32.3–38.5 mm.^26^ The pulp cavity is broadly open at a newborn stage and it closes gradually during the first 4–5 years of life, until only a narrow canal for the blood vessels remains open.^26^ Both the grey seal canines (the modern sample 004 and sample 015 from Stora Förvar) were estimated to belong to seals younger than 3 months, as they measured 26 and 25 mm, respectively, and both had broadly open pulp cavities (8 and 9 mm, respectively). The grey seal molar from Stora Förvar (sample 013) was not possible to age, since there are no ageing data on molars in grey seals, but the fact that the pulp cavity is closed suggests that the seal was older than 12 months (Table 1).

Ringed seals are born with permanent canines^27^ and tooth eruption completes shortly after birth. The canine measured 19 mm, which, together with the closed pulp cavity, suggests an adult animal.^28^

### 2.3 Strontium and incremental growth in tooth enamel

Strontium becomes incorporated into hydroxyapatite of tooth enamel during the mineralization process of the enamel. This happens through interactions between Ca and Sr ions which result in exchange of ions and substitution of Ca ions by Sr ions from the surfaces of hydroxyapatite crystals.^29^ This occurs because [Sr]^2+^ has similar physical and chemical properties to [Ca]^2+^, its closest neighbor among the alkaline earth metals in the Periodic Table.^29^, ^30^, ^31^ Strontium in tooth enamel is resistant to taphonomic diagenesis, in contrast to strontium in bones and dentine,^32^, ^33^ and is therefore suitable for analysis.

Enamel formation, or amelogenesis, is a prolonged and slow process which consists of two main stages: the secretory phase and the maturation phase.^34^, ^35^, ^36^ Enamel formation is controlled and regulated by the ameloblasts, enamel‐forming cells, which enable enamel matrix secretion and deposition from the tooth crown towards the cervix. At this stage the enamel consists mainly of organic matter (proteins). During enamel maturation, organic matter and water are removed and replaced by inorganic matter, forming apatite crystals, which grow, eventually leading to final enamel hardening. The mineralization of the enamel can be divided into two main stages – pre‐ and post‐eruptive mineralization. The pre‐eruptive enamel mineralization takes place when the ameloblasts, after the initial secretion of protein matrix, switch their function to active mineral deposition. The post‐eruptive mineralization takes place at the outermost enamel layer and is mediated by mineral ions and other components obtained by the saliva.^35^, ^37^, ^38^

Tooth enamel is suitable for intra‐tooth sequential sampling because after its formation there is no remodeling of the tissue. Thus, by analyzing the isotopic composition of enamel, it is possible to study mobility, dietary and climatic factors sequentially from the whole duration of its formation.

The deciduous dentition in phocid seals is shed *in utero* and the permanent teeth are formed prior to birth. The enamel of the permanent dentition is laid down before birth.^13^, ^39^ Specifically, harp seal canines are fully erupted within the first week after birth, grey seal canines erupt 3–4 weeks after birth and in ringed seals the canines erupt shortly after birth. This implies that the pre‐eruptive maturation of the enamel has been completed in all three species while they are still fetuses. By and large, the time elapsed between birth and until the canines are fully erupted coincides in harp and grey seals with their estimated lactation period, while for ringed seals the lactation period lasts longer. Consequently, seal pups in all three species are still incorporating maternal isotopic ratios until the canines are fully erupted. Thus, in terms of biological age, the sequential sampling of the tooth enamel from the tip of the crown towards the cervix is expected to reflect the last months of the fetus prior to birth. In other words, it reflects the mother's isotopic ratios during the last months of gestation.

By calculating the duration from the time that the permanent dentition is formed *in utero* until the full eruption of the canine after birth, it is possible to narrow down the time that the sequential sampling reflects in terms of biological age. Data from modern harp seals show that the permanent dentition is already formed *in utero* around December^23^ and these seals give birth in March. Thus, the sequential sampling of the enamel should reflect approximately the last 3 months of gestation. In ringed seals, the permanent dentition forms in November^27^ approximately 4 months before birth. No data are available for the prenatal dental ontogeny of grey seals but we assume a similar development to that of the other two species. Thus, the sequential sampling for all three species should reflect a time span of approximately 3–4 months prior to birth.

Little is known regarding the duration of the post‐eruptive maturation and how that affects the Sr isotope ratios in tooth enamel. For some species the duration of the post‐eruptive maturation has been determined: 2 weeks for rats;^34^ several months for cattle;^40^ and up to several years for humans.^35^ For pinnipeds the time elapsed from deposition of the enamel matrix to completion of the post‐eruptive mineralization is not known.

Even if we calculated a prolonged post‐eruptive mineralization of at least some weeks, in which Sr ions would enter the enamel hydroxyapatite during mineralization, it would remain questionable to what extent this process affects the Sr isotopic ratios in tooth enamel. Therefore, for this study we assume that the isotopic ratios reflect only the mother's dietary and mobility patterns during gestation.

Although intra‐tooth variation in enamel analysis might be biased by the pattern and duration of enamel mineralization, oxygen isotope analyses on enamel apatite from herbivores have shown that the sequential sampling of enamel reflects a chronological sequence, which makes it suitable for detecting seasonal variations.^40^, ^41^ Thus, sequential sampling of teeth can provide insights into certain environmental events, diet and mobility.

### 2.4 Sample preparation

The surface of the teeth was gently cleaned with a brush and deionized water. The samples were then sonicated in deionized water for approximately 1 min and subsequently rinsed with deionized water; this procedure was repeated twice. The samples were then left to dry at room temperature. Before laser ablation analysis was performed, the enamel surface was cleaned with ethanol.

### 2.5 Horizontal sequential sampling

Sr isotope ratios of enamel were measured *in situ* on the teeth at the Vegacenter facility at the Swedish Museum of Natural History (Stockholm, Sweden) using a NWR193 excimer laser ablation system (Electro Scientific Industries, Portland, OR, USA) coupled to a Nu Plasma II multi‐collector ICP mass spectrometer (Nu Instruments Ltd, Wrexham, UK). Nitrogen was introduced using an interconnected Aridus II desolvating nebulizer system (Teledyne Cetac, Omaha, NE, USA). The instrument operating conditions are listed in Table S1 (supporting information). A sample cell with a flexible mounting table allows insertion of the specimens without embedding or cutting them. After cleaning, the teeth were mounted as parallel as possible to the cell surface. Prior to analysis, tracks perpendicular to the direction of growth of the tooth were pre‐ablated using a spot size of 150 μm to remove potential surface contamination. Tracks of 650 to 900 μm length using a 140 μm spot size were then ablated on the pre‐ablated enamel surface. The number of ablation lines per tooth ranged from 14 to 40, depending on the tooth size.

Possible isobaric interferences of krypton were measured for 30 s in the gas baseline before the analysis and subtracted from the collected masses. Doubly charged rare earth elements (REE) are measured on less interfered masses (^163^Dy^2+^ on 81.5, ^166^Er^2+^ on 83 and ^173^Yb^2+^ on 86.5) and subtracted from the Sr masses as well as from the signal on mass 82. Mass 82 allows for the assessment of the contribution of Ca‐dimers and Ca‐argides on the Sr isotope ratios. After these corrections are applied, a fractionation factor is calculated based on the stable ^86^Sr/^88^Sr ratio and is used to quantify the contribution of Rb to *m/z* 87 (stable ^87^Rb/^85^Rb is assumed to be 0.3861). All corrections are applied online to each integration allowing interference‐ and fractionation‐corrected Sr ratios to be determined for every data point (Table 2).

**Table 2:** Collector configuration on the Nu Plasma II (Vegacenter, Swedish Museum of Natural History), list of possible interferences in order of applied corrections and type of correction (baseline subtraction: baseline recorded for 30 seconds before each ablation, peak stripping: interference measured on least interfered mass and then calculated for other isotopes using their natural abundance, low oxide tuning: see description in text)

Mass		81.5	82	83	84	85	86	86.5	87	88
Faraday cup		L5	L4	L2	Ax	H2	H4	H5	H6	H8
Strontium					^84^Sr^+^		^86^Sr^+^		^87^Sr^+^	^88^Sr^+^
Krypton	baseline subtraction		^82^Kr^+^	^83^Kr^+^	^84^Kr^+^		^86^Kr^+^			
REE^2+^	peak stripping	^163^Dy^2+^	(^164^Er^2+^) ^164^Dy^2+^	^166^Er^2+^	^168^Er^2^ (^168^Yb^2+^)	^170^Er^2+^ (^170^Yb^2+^)	^172^Yb^2+^	^163^Yb^2+^	^174^Yb^2+^	^176^Yb^2+^
Ca‐dimers	peak stripping		^42^Ca^40^Ca^+^	^43^Ca^40^Ca^+^	^44^Ca^40^Ca^+^	^42^Ca^43^Ca^+^	^46^Ca^40^Ca^+^			^48^Ca^40^Ca^+^
Ca‐argides	peak stripping		^42^Ca^40^Ar^+^	^43^Ca^40^Ar^+^	^44^Ca^40^Ar^+^		^46^Ca^40^Ar^+^			^48^Ca^40^Ar^+^
Rubidium	peak stripping					^85^Rb^+^			^87^Rb^+^	
^40^ (Ca/Ar)^31^P^16^O^+^	low oxide tuning						^40^ (Ca/Ar)^31^P^16^O^+^			

An additional polyatomic interference on *m/z* 87 has been reported (e.g. 42, 43) and is described as 40 (Ca/Ar)^31^P^16^O^+^. This interference can introduce a significant offset in ^87^Sr/^86^Sr, especially for samples with low Sr concentrations. This interference cannot be corrected online but has to be reduced by a thorough low oxide tuning of the gases.^43^ We achieve this through the introduction of nitrogen (~8 mL/min) into the He‐Ar‐Sample mixture prior to injection into the plasma using a CETAC Aridus II, while simultaneously aspirating pure 0.3 M nitric acid. This allows UO^+^/U^+^ oxide production to be kept well below 1%.

To verify that all interferences are successfully removed, a tooth reference material (Otomys 26‐r52^44^) is measured three times before and after a sample is measured (an experiment comprises typically 15–40 line scans on one specimen). The average of 37 analyses during the measurement session yielded a ^87^Sr/^86^Sr ratio of 0.72050 ± 0.00054, which agrees well with the value determined by thermal ionization mass spectrometry (0.720525 ± 0.000090; Finnigan Triton, Thermo Scientific, Waltham, MA, USA) at the Swedish Museum of Natural History (Stockholm, Sweden).

## 3 RESULTS

**Figure 2:**
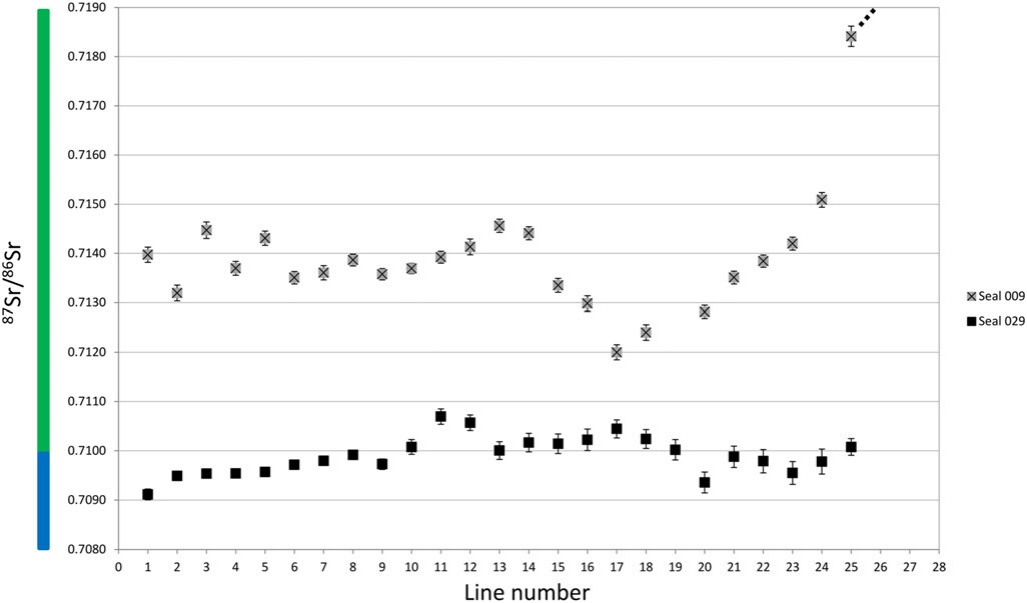
^87^Sr/^86^Sr ratios for harp seal teeth. The x‐axis represents the sampling lines from the tip of the tooth (1) towards the cervix (26). Dotted line indicates a higher value which is displayed in Figure 5. Error bars are the external precision 2SD (see Table S2, supporting information) [Color figure can be viewed at http://wileyonlinelibrary.com]

**Figure 3:**
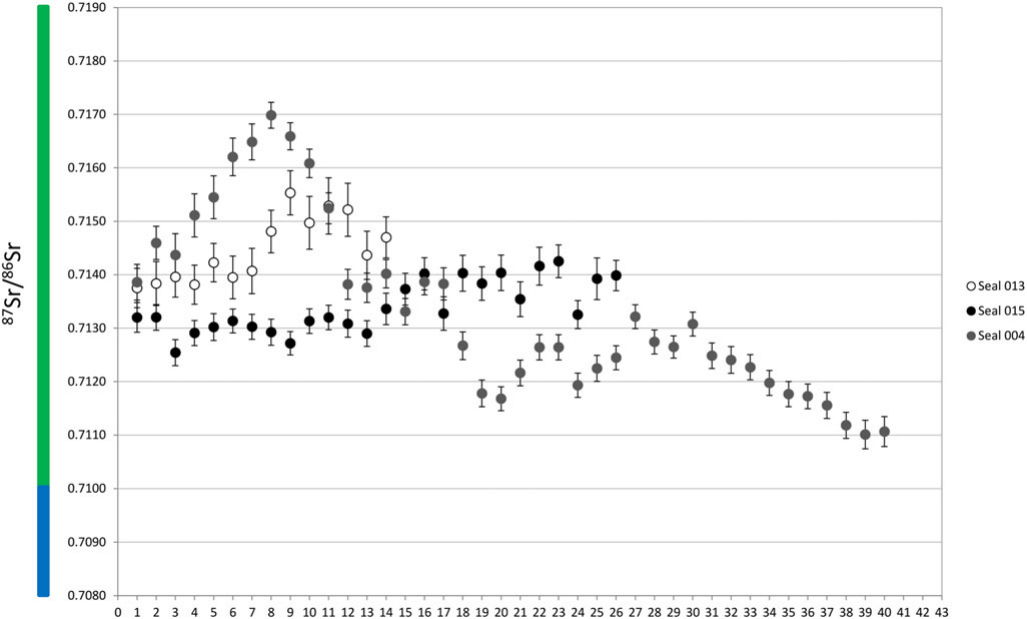
^87^Sr/^86^Sr ratios for grey seal teeth. The x‐axis represents the sampling lines from the tip of the tooth (1) towards the cervix (40). Error bars are the external precision 2SD (see Table S2, supporting information) [Color figure can be viewed at http://wileyonlinelibrary.com]

**Figure 4:**
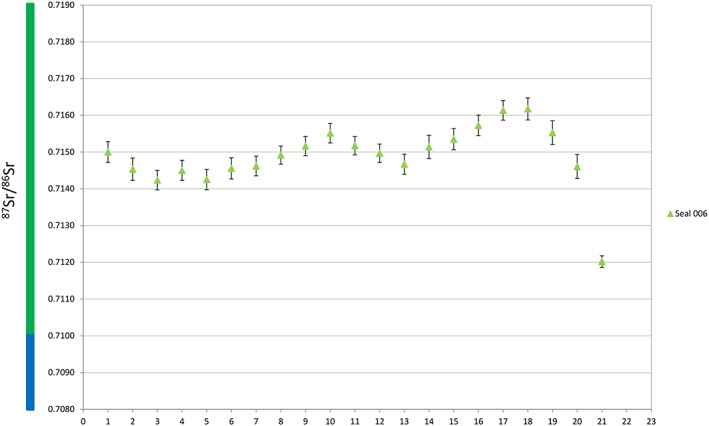
^87^Sr/^86^Sr ratios for ringed seal tooth. The x‐axis represents the sampling lines from the tip of the tooth (1) towards the cervix (21). Error bars are the external precision 2SD (see Table S2, supporting information) [Color figure can be viewed at http://wileyonlinelibrary.com]

To facilitate interpretation, we have colour‐coded the ^87^Sr/^86^Sr ratios in five intervals in plots and in the map (Figures 1, 2, 3, 4). Accordingly, oceanic water and other ratios <0.7100 are blue, ratios 0.7100–0.7199 are green, 0.7200–0.7299 are yellow, 0.7300–0.7399 are orange, and ≥ 0.740 are red.

The harp seal canine from Ajvide (sample 029, Figures 2 and 6) yielded data that show various small‐scale fluctuations in the ^87^Sr/^86^Sr ratio between ~0.7091 and ~0.7107 (between blue and green). The measurement distance between each of the first four lines (Figure 6) was much larger than the rest (700 μm vs 200 μm – this was the first tooth that we analyzed and we did not know what kind of variation to expect; see also Table S3, supporting information). The first measurement (below the canine tip) yielded a ^87^Sr/^86^Sr ratio of 0.70912 (blue), which is close to the oceanic Sr ratio (0.70918) and to the ^87^Sr/^86^Sr ratios of water samples from Skagerrak (0.70917), Kattegat (0.70920) and the Baltic Proper (0.70921–0.70932)^4^ (for measurement uncertainties, see Table S2, supporting information). Measurements 2–9 (blue) show a gradual increase, until at measurement 10 they enter the green interval, which corresponds to the discharge areas of rivers into the southern Baltic (Table S2, supporting information; Figure 1). Measurements 20–24 (blue) show a slight decrease, whereas the last measurement increases slightly to 0.71008 (green).

The harp seal canine from Korsnäs (sample 009, Figures 2 and 7) yielded ^87^Sr/^86^Sr ratios that were all higher than those of the Ajvide sample, and show much larger fluctuations; the total range is ~0.7120–0.7241 (from green to yellow). Measurements 27–29 are omitted because they were made on dentine (Table S2, supporting information; Figure 2). Measurements 1–23 fluctuate between ~0.7120 and ~0.7146 (green), which is almost twice the range for the Ajvide sample. In contrast, measurements 24–26 show a very drastic change with Sr ratios ranging from ~0.7151 to ~0.7241 (from green to yellow).

The grey seal canine from Stora Förvar (sample 015, Figure 3; Table S2, supporting information) yielded ^87^Sr/^86^Sr ratios that fluctuated from ~0.7125 to ~0.7142 (green). The profile can be roughly divided into two groups – one closest to the tip of the tooth ^87^Sr/^86^Sr ratios of 0.71300 ± 0.00040 (*n* = 13, measurements 1–13) and a second one with a higher range of ^87^Sr/^86^Sr ratios, on average 0.71380 ± 0.00068 (n = 13, measurements 14–26).

A grey seal molar (sample 013, Figure 3; Table S2, supporting information) from Stora Förvar yielded small‐scale fluctuations in the ^87^Sr/^86^Sr ratio between ~0.7138 and ~0.7155 (green). The first seven sampling lines yield ratios that fluctuate around 0.7139, whereas the measurements from the lower half of the tooth oscillate around 0.7150, with a slightly more scattered distribution.

The canine from a modern grey seal from the Baltic Sea (sample 004, Figure 3; Table S2, supporting information) shows ^87^Sr/^86^Sr ratios between ~0.7111 and ~0.7170 (green). The first eight measurements show a steady increase, from ~0.7139 to ~0.7170, followed by a continuous decrease, although with fluctuations, reaching the lowest value with the last measurement.

The ringed seal canine (sample 006, Figure 4; Table S2, supporting information) from Stora Förvar displayed a ^87^Sr/^86^Sr range between ~0.7142 and ~0.7162 (green).

## 4 DISCUSSION

All the individuals in this study were sampled in the same way, and the study compares individuals from species of the same family, taking into account any species‐dependent differences in the pattern and duration of enamel mineralization that might cause biases regarding interpretation.^41^

**Figure 5:**
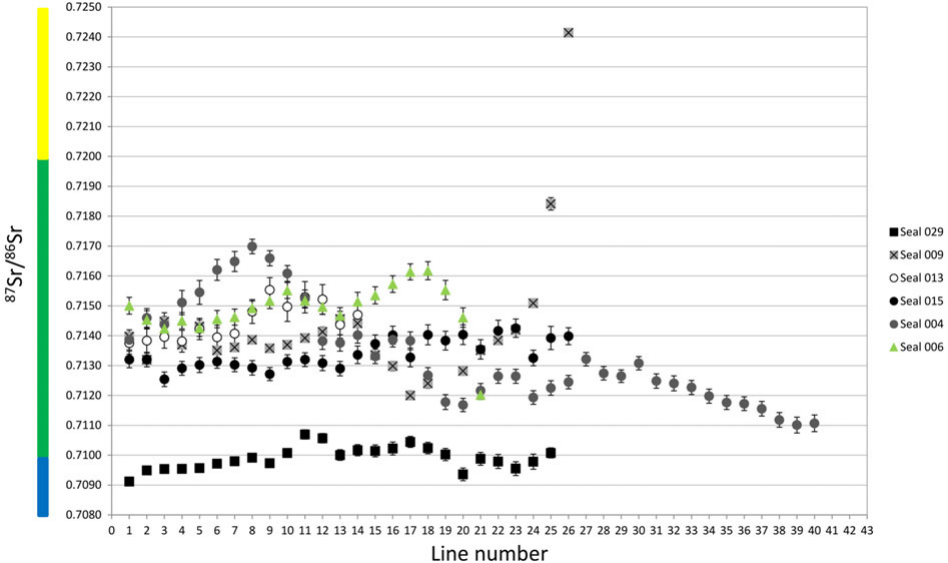
^87^Sr/^86^Sr ratios for all seal teeth. Error bars are the external precision 2SD (see Table S2, supporting information). Legend: circles: grey seals; squares: harp seals; triangles: ringed seal [Color figure can be viewed at http://wileyonlinelibrary.com]

Harp seals are nowadays highly migratory. They start their annual migration northwards as soon as the ice clears. In general harp seals stay close to pack ice all year round and return to their breeding grounds in late autumn to give birth. The two harp seal canines that were analyzed in this study yielded isotope ratios that were significantly different from each other (Figures 2 and 5). The first measurement of sample 029 from Ajvide, just below the tip (^87^Sr/^86^Sr = 0.70912), yielded a ratio corresponding to the oceanic ^87^Sr/^86^Sr ratio, which might indicate foraging either in the Atlantic or in the Skagerrak/Kattegat areas. Because the enamel formation and main maturation of the permanent dentition occur in pinnipeds during gestation, the Sr ratios should reflect the movements of the pregnant harp seal during the last months of gestation when the permanent dentition forms *in utero*. Accordingly, the female seal might have been foraging in the Atlantic Ocean close to the Skagerrak/Kattegat areas and then moved into the Baltic Proper. All measurements, except for the first one, yielded Sr ratios corresponding to Baltic Sea water ratios or to water areas where rivers discharge into the Baltic Sea. Thus, parturition occurred in the Baltic Sea, confirming that harp seals had their breeding grounds in the Baltic Sea region. The last Sr ratios are slightly elevated and may reflect seasonal movements within the southern Baltic, or theoretically to the Bothnian Sea. Considering that the canine belonged to a very young harp seal, younger than 6 months, it is possible that the young seal died before its first migration.

**Figure 6:**
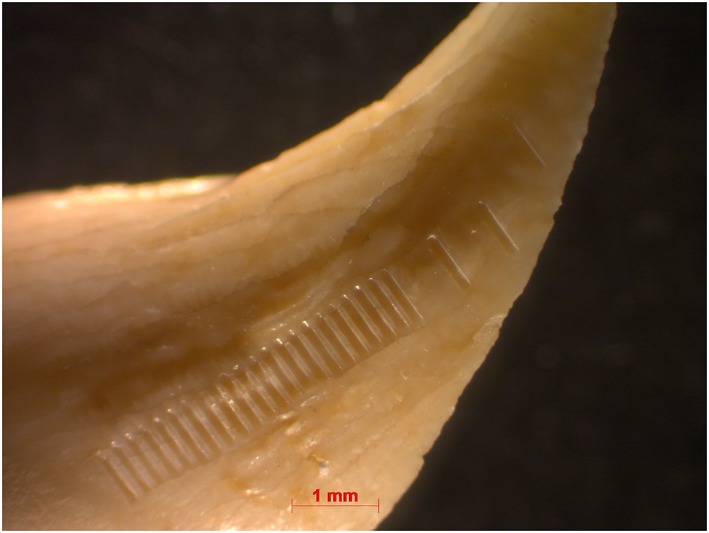
Harp seal canine (seal 029) with sampling lines. Distance between lines is 700 μm for lines 1–4 and 200 μm for lines 4–25 [Color figure can be viewed at http://wileyonlinelibrary.com]

**Figure 7:**
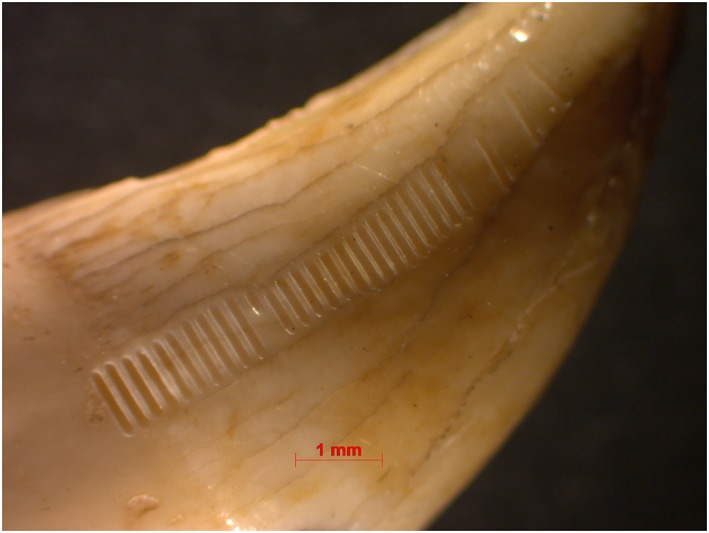
Harp seal canine (seal 009) with sampling lines. Distance between lines is 600 μm for lines 1–4 and 200 μm for lines 4–29. Sampling lines 28–29 are on dentine, sampling line 27 is on the dentine‐enamel transition. Line 19 was interrupted because of machine failure [Color figure can be viewed at http://wileyonlinelibrary.com]

The second harp seal, sample 009 from Korsnäs, reveals a different pattern. All Sr ratios correspond to Baltic Sea values (Figures 2 and 5). The first 23 measurements indicate that the pregnant seal was foraging in the southwestern Baltic Sea possibly reflecting seasonal foraging movements to the discharge areas of the rivers, even foraging in the river mouth, where we assume that the Sr ratios of the waters are closer to the riverine ^87^Sr/^86^Sr ratio. A significant increase in ^87^Sr/^86^Sr in the lower half of the tooth indicates a movement within the Baltic Sea either towards the northernmost part of the Bothnian Bay or towards the Gulf of Finland. The significant change in the ratios might be linked to seasonal migration patterns of the harp seal.

Even if there are only two harp seal individuals, it is still possible to retrieve information on potential migration and breeding patterns of this now extinct species in the Baltic Sea. The data suggest there might be two different behavioral patterns: one harp seal was foraging in the Atlantic Ocean during gestation and entered the Baltic Sea where it gave birth, and the other seal spent its entire life in the Baltic Sea. Since pregnant harp seals spend most time of their gestation while migrating, this would indicate either a migration route from the Baltic Sea to the Atlantic Ocean and then back to its breeding ground in the Baltic Sea, or that the harp seal belonged to an Atlantic harp seal population and migrated into the Baltic Sea where parturition took place. Intrusions into the Baltic Sea of different marine mammals have been recorded several times in recent years. However, the latter scenario seems to be less probable since osteometrical analysis has shown that the Baltic harp seals had a smaller body size than the Atlantic harp seal population.^15^ In the other harp seal, by contrast, there is no indication that it spent time in the Atlantic or Kattegat/Skagerrak area. Whether the two harp seals belonged to different populations with distinct behavioural patterns is a very intriguing question resulting from this study that warrants further investigation.

Both grey seals and ringed seals are non‐migratory and thus it was not expected that their ^87^Sr/^86^Sr ratios would vary substantially. The two grey seals from Stora Förvar (samples 013 and 015; Figure 5) demonstrated some intra‐tooth variations, indicating seasonal movements to water sources with slightly different Sr ratios. This suggests foraging in river mouths, perhaps in search of Salmonidae, one of the main preferable prey for grey seals.^9^, ^45^ The small Sr isotope variations suggest a very consistent foraging pattern in these individuals, consistent with a rather philopatric behavior. The modern canine displays values that indicate movements more or less in the same geographic area as the other two measured grey seals, but the extent of the movements is larger.

This is in accordance with the life pattern of grey seals as they are non‐migratory but can undertake long foraging trips in search of food, as evidenced by the larger variation in ^87^Sr/^86^Sr ratios in the canine from the modern grey seal (sample 004). Recent satellite tracking studies also demonstrate that grey seals can cover long distances within the Baltic Sea in search of food.^11^, ^12^ All three grey seal samples indicated foraging in the southwestern Baltic Sea. This is in accordance with the known distribution of the modern grey seal population in the Baltic Sea which has its westernmost distribution around Bornholm.^46^

The ringed seal has ^87^Sr/^86^Sr ratios within the same range as the grey seal, indicating a similar geographic distribution in the southwestern Baltic Sea and possible seasonal movements between water sources in the same region as the grey seals. This is in sharp contrast to the present‐day distributions in the Baltic Sea, where ringed seals mainly occur in the Gulf of Bothnia, the Gulf of Finland and the Gulf of Riga, where the ice conditions allow proper breeding grounds.^47^

## 5 CONCLUSIONS

This study demonstrates that ^87^Sr/^86^Sr ratios in some marine environments such as the Baltic are not necessarily uniform. Differences in ^87^Sr/^86^Sr ratios can therefore be detected in marine mammals to study migration patterns. Further analysis should be performed in order to identify regional differences within the Baltic Sea basin at a more detailed level.

The results of this study also have implications for the archaeological interpretation of human mobility or migration. Strontium in tooth enamel has been used as an indicator of human mobility between regions with different Sr isotope ratios. Our study shows that for prehistoric coastal cultures, especially in regions with freshwater influx such as the Baltic Sea, where marine mammal hunting and consumption have been significant for human subsistence, such an interpretation should be applied with caution. We suggest that the differences in ^87^Sr/^86^Sr ratios between different regions should be reflected in humans, as consumers of seals and fish, and thus interpretation of such data should be ideally performed in combination with carbon and nitrogen isotope analysis as indicators for diet.

## Supporting information


**Supplementary Table S1.** Operating conditions for LA‐MC‐ICP‐MS Sr isotope measurements
**Supplementary Table S2.** All ^87^Sr/^86^Sr measurements for each sample
**Supplementary Table S3.** Space in μm between the sampling lines and sampling time for each sample
