# Supplementary material for: Intra‐ and inter‐tooth variation in strontium isotope ratios from prehistoric seals by laser ablation multi‐collector inductively coupled plasma mass spectrometry
Source: Rapid Commun Mass Spectrom. 2018 Jun 26;32(15):1215–24. doi: 10.1002/rcm.8158 (PMC6033018; doi:10.1002/rcm.8158)
Supplement: Supplementary file 1 — Supplementary Table S1. Operating conditions for LA‐MC‐ICP‐MS Sr isotope measurements Supplementary Table S2. All 87Sr/86Sr measurements for each sample Supplementary Table S3. Space in μm between the sampling lines and sampling time for each sample [file RCM-32-1215-s001.docx]

Supplementary Table 1. Operating conditions for LA-MC-ICP-MS Sr isotope measurements.

| Mass spectrometer | Nu Plasma II MC-ICP mass spectrometer |
| --- | --- |
| Cooling gas (Ar) flow rate | 13 L/min |
| Aux gas (Ar) flow rate | 0.95 L/min |
| Mass resolution | ~1200 (M/ΔM) |
| Laser ablation | ESI NWR193 ArF excimer laser ablation system |
| Ar flow rate (Mix Gas) | 0.25 - 0.55 L/min |
| He flow rate | 0.30 - 0.32 L/min |
|  | |
| *Pre-ablation* | |
| Frequency | 3 Hz |
| Translation rate | 50 μm/s |
| Spot size | 150 μm |
| Fluence | 0.5 J/cm^2^ |
|  | |
| *Ablation* | |
| Frequency | 25 Hz |
| Translation rate | 5 μm/s |
| Spot size | 140 μm |
| Fluence | 1.7 - 2.1 J/cm^2^ |
|  | |
| *Data collection* | |
| Gas baseline | 30 s |
| Sample | 90 - 160 s |
| Nitrogen introduction | CETAC Aridus II desolvating nebulizer system |
| N_2_ flow rate | 8 mL/min |
| Nebuliser pressure (Ar) | 22.2-23.5 psi |
| Ar sweep gas | 1.2 - 1.4 L/min |
| Aspiration | 0.3 M HNO_3_ |

Supplementary Table 2. All ^87^Sr/^86^Sr measurements for each sample. *propagated uncertainty calculated by multiplying 2SE of unknowns with 2SD/2SE_average_ of the rodent standard measurements.

| **Sample** | **line number** | **^87^Sr/^86^Sr** | **internal precision 2SE** | **external precision 2SD*** | **total Sr (V)** |
| --- | --- | --- | --- | --- | --- |
| 029 |  |  |  |  |  |
|  | 1 | 0.70912 | 0.00008 | 0.00011 | 1.49 |
|  | 2 | 0.70949 | 0.00007 | 0.00009 | 1.83 |
|  | 3 | 0.70954 | 0.00007 | 0.00009 | 1.88 |
|  | 4 | 0.70954 | 0.00007 | 0.00009 | 1.98 |
|  | 5 | 0.70957 | 0.00007 | 0.00009 | 2.08 |
|  | 6 | 0.70972 | 0.00006 | 0.00008 | 2.45 |
|  | 7 | 0.70980 | 0.00007 | 0.00010 | 2.37 |
|  | 8 | 0.70992 | 0.00006 | 0.00009 | 2.24 |
|  | 9 | 0.70973 | 0.00008 | 0.00011 | 2.06 |
|  | 10 | 0.71008 | 0.00011 | 0.00015 | 1.47 |
|  | 11 | 0.71069 | 0.00012 | 0.00016 | 1.41 |
|  | 12 | 0.71057 | 0.00012 | 0.00016 | 1.24 |
|  | 13 | 0.71001 | 0.00013 | 0.00018 | 1.07 |
|  | 14 | 0.71017 | 0.00014 | 0.00019 | 0.97 |
|  | 15 | 0.71014 | 0.00015 | 0.00020 | 0.96 |
|  | 16 | 0.71022 | 0.00016 | 0.00022 | 0.80 |
|  | 17 | 0.71044 | 0.00014 | 0.00018 | 0.96 |
|  | 18 | 0.71024 | 0.00014 | 0.00019 | 0.90 |
|  | 19 | 0.71002 | 0.00015 | 0.00021 | 0.74 |
|  | 20 | 0.70936 | 0.00016 | 0.00021 | 0.68 |
|  | 21 | 0.70988 | 0.00016 | 0.00022 | 0.68 |
|  | 22 | 0.70979 | 0.00017 | 0.00024 | 0.63 |
|  | 23 | 0.70955 | 0.00017 | 0.00023 | 0.57 |
|  | 24 | 0.70978 | 0.00019 | 0.00025 | 0.62 |
|  | 25 | 0.71008 | 0.00012 | 0.00017 | 1.18 |
| 009 |  |  |  |  |  |
|  | 1 | 0.71397 | 0.00011 | 0.00015 | 2.90 |
|  | 2 | 0.71320 | 0.00012 | 0.00016 | 3.57 |
|  | 3 | 0.71447 | 0.00012 | 0.00017 | 4.46 |
|  | 4 | 0.71370 | 0.00010 | 0.00014 | 4.11 |
|  | 5 | 0.71431 | 0.00011 | 0.00015 | 3.74 |
|  | 6 | 0.71351 | 0.00009 | 0.00013 | 4.35 |
|  | 7 | 0.71361 | 0.00011 | 0.00014 | 4.68 |
|  | 8 | 0.71387 | 0.00009 | 0.00012 | 4.54 |
|  | 9 | 0.71358 | 0.00008 | 0.00011 | 3.69 |
|  | 10 | 0.71369 | 0.00008 | 0.00010 | 3.48 |
|  | 11 | 0.71392 | 0.00009 | 0.00012 | 3.31 |
|  | 12 | 0.71413 | 0.00012 | 0.00016 | 3.25 |
|  | 13 | 0.71456 | 0.00010 | 0.00013 | 3.22 |
|  | 14 | 0.71441 | 0.00010 | 0.00013 | 3.29 |
|  | 15 | 0.71335 | 0.00010 | 0.00014 | 2.85 |
|  | 16 | 0.71298 | 0.00012 | 0.00016 | 2.62 |
|  | 17 | 0.71200 | 0.00011 | 0.00015 | 2.87 |
|  | 18 | 0.71240 | 0.00012 | 0.00016 | 2.66 |
|  | 19 | missing due to instrument failure | | | |
|  | 20 | 0.71282 | 0.00010 | 0.00013 | 2.68 |
|  | 21 | 0.71351 | 0.00010 | 0.00013 | 2.75 |
|  | 22 | 0.71384 | 0.00009 | 0.00012 | 2.89 |
|  | 23 | 0.71420 | 0.00010 | 0.00013 | 2.90 |
|  | 24 | 0.71509 | 0.00011 | 0.00015 | 2.49 |
|  | 25 | 0.71841 | 0.00015 | 0.00021 | 2.78 |
|  | 26 | 0.72414 | 0.00011 | 0.00014 | 4.35 |
| *dentine* | *27* | *0.72732* | *0.00008* | *0.00010* | *7.23* |
| *dentine* | *28* | *0.72784* | *0.00007* | *0.00009* | *7.81* |
| *dentine* | *29* | *0.72791* | *0.00009* | *0.00013* | *7.46* |
| 015 |  |  |  |  |  |
|  | 1 | 0.71320 | 0.00020 | 0.00028 | 0.61 |
|  | 2 | 0.71320 | 0.00018 | 0.00024 | 0.60 |
|  | 3 | 0.71254 | 0.00018 | 0.00024 | 0.68 |
|  | 4 | 0.71291 | 0.00017 | 0.00023 | 0.71 |
|  | 5 | 0.71302 | 0.00019 | 0.00025 | 0.74 |
|  | 6 | 0.71314 | 0.00017 | 0.00022 | 0.72 |
|  | 7 | 0.71303 | 0.00017 | 0.00023 | 0.77 |
|  | 8 | 0.71292 | 0.00018 | 0.00024 | 0.73 |
|  | 9 | 0.71272 | 0.00016 | 0.00022 | 0.74 |
|  | 10 | 0.71313 | 0.00017 | 0.00023 | 0.77 |
|  | 11 | 0.71320 | 0.00017 | 0.00023 | 0.74 |
|  | 12 | 0.71308 | 0.00019 | 0.00025 | 0.69 |
|  | 13 | 0.71290 | 0.00018 | 0.00024 | 0.69 |
|  | 14 | 0.71336 | 0.00022 | 0.00030 | 0.69 |
|  | 15 | 0.71373 | 0.00022 | 0.00030 | 0.67 |
|  | 16 | 0.71402 | 0.00022 | 0.00030 | 0.66 |
|  | 17 | 0.71327 | 0.00023 | 0.00031 | 0.62 |
|  | 18 | 0.71403 | 0.00025 | 0.00034 | 0.60 |
|  | 19 | 0.71383 | 0.00023 | 0.00031 | 0.59 |
|  | 20 | 0.71404 | 0.00024 | 0.00033 | 0.58 |
|  | 21 | 0.71354 | 0.00024 | 0.00032 | 0.62 |
|  | 22 | 0.71416 | 0.00026 | 0.00035 | 0.62 |
|  | 23 | 0.71425 | 0.00023 | 0.00031 | 0.64 |
|  | 24 | 0.71325 | 0.00019 | 0.00026 | 0.65 |
|  | 25 | 0.71392 | 0.00029 | 0.00039 | 0.56 |
|  | 26 | 0.71398 | 0.00021 | 0.00028 | 0.62 |
| 013 |  |  |  |  |  |
|  | 1 | 0.71375 | 0.00027 | 0.00037 | 0.31 |
|  | 2 | 0.71384 | 0.00030 | 0.00041 | 0.31 |
|  | 3 | 0.71396 | 0.00028 | 0.00038 | 0.30 |
|  | 4 | 0.71381 | 0.00027 | 0.00037 | 0.28 |
|  | 5 | 0.71423 | 0.00027 | 0.00036 | 0.30 |
|  | 6 | 0.71395 | 0.00030 | 0.00040 | 0.27 |
|  | 7 | 0.71407 | 0.00031 | 0.00042 | 0.27 |
|  | 8 | 0.71481 | 0.00029 | 0.00040 | 0.29 |
|  | 9 | 0.71553 | 0.00031 | 0.00041 | 0.25 |
|  | 10 | 0.71497 | 0.00036 | 0.00049 | 0.23 |
|  | 11 | 0.71529 | 0.00039 | 0.00053 | 0.22 |
|  | 12 | 0.71521 | 0.00037 | 0.00050 | 0.22 |
|  | 13 | 0.71436 | 0.00033 | 0.00045 | 0.24 |
|  | 14 | 0.71470 | 0.00028 | 0.00038 | 0.27 |
| 004 |  |  |  |  |  |
|  | 1 | 0.71386 | 0.00025 | 0.00034 | 0.93 |
|  | 2 | 0.71459 | 0.00023 | 0.00031 | 0.90 |
|  | 3 | 0.71437 | 0.00029 | 0.00040 | 0.75 |
|  | 4 | 0.71511 | 0.00030 | 0.00040 | 0.80 |
|  | 5 | 0.71545 | 0.00030 | 0.00040 | 0.70 |
|  | 6 | 0.71620 | 0.00026 | 0.00035 | 0.68 |
|  | 7 | 0.71649 | 0.00025 | 0.00034 | 0.67 |
|  | 8 | 0.71698 | 0.00018 | 0.00024 | 0.63 |
|  | 9 | 0.71659 | 0.00019 | 0.00025 | 0.57 |
|  | 10 | 0.71608 | 0.00020 | 0.00027 | 0.53 |
|  | 11 | 0.71524 | 0.00021 | 0.00029 | 0.49 |
|  | 12 | 0.71382 | 0.00021 | 0.00028 | 0.47 |
|  | 13 | 0.71376 | 0.00020 | 0.00027 | 0.49 |
|  | 14 | 0.71402 | 0.00020 | 0.00027 | 0.52 |
|  | 15 | 0.71331 | 0.00018 | 0.00025 | 0.58 |
|  | 16 | 0.71386 | 0.00018 | 0.00024 | 0.59 |
|  | 17 | 0.71383 | 0.00022 | 0.00030 | 0.52 |
|  | 18 | 0.71267 | 0.00019 | 0.00026 | 0.53 |
|  | 19 | 0.71178 | 0.00018 | 0.00025 | 0.55 |
|  | 20 | 0.71168 | 0.00016 | 0.00022 | 0.54 |
|  | 21 | 0.71216 | 0.00018 | 0.00024 | 0.57 |
|  | 22 | 0.71264 | 0.00017 | 0.00024 | 0.58 |
|  | 23 | 0.71264 | 0.00017 | 0.00023 | 0.55 |
|  | 24 | 0.71193 | 0.00017 | 0.00023 | 0.54 |
|  | 25 | 0.71225 | 0.00018 | 0.00024 | 0.55 |
|  | 26 | 0.71245 | 0.00017 | 0.00023 | 0.56 |
|  | 27 | 0.71321 | 0.00017 | 0.00022 | 0.56 |
|  | 28 | 0.71274 | 0.00017 | 0.00022 | 0.57 |
|  | 29 | 0.71265 | 0.00015 | 0.00021 | 0.59 |
|  | 30 | 0.71308 | 0.00016 | 0.00022 | 0.55 |
|  | 31 | 0.71248 | 0.00018 | 0.00024 | 0.52 |
|  | 32 | 0.71240 | 0.00018 | 0.00025 | 0.51 |
|  | 33 | 0.71227 | 0.00017 | 0.00024 | 0.48 |
|  | 34 | 0.71198 | 0.00017 | 0.00023 | 0.49 |
|  | 35 | 0.71177 | 0.00017 | 0.00023 | 0.50 |
|  | 36 | 0.71172 | 0.00017 | 0.00023 | 0.52 |
|  | 37 | 0.71156 | 0.00018 | 0.00024 | 0.46 |
|  | 38 | 0.71118 | 0.00018 | 0.00024 | 0.46 |
|  | 39 | 0.71101 | 0.00020 | 0.00027 | 0.40 |
|  | 40 | 0.71107 | 0.00021 | 0.00028 | 0.41 |
| 006 |  |  |  |  |  |
|  | 1 | 0.71500 | 0.00021 | 0.00028 | 0.42 |
|  | 2 | 0.71453 | 0.00023 | 0.00031 | 0.43 |
|  | 3 | 0.71423 | 0.00020 | 0.00026 | 0.43 |
|  | 4 | 0.71450 | 0.00020 | 0.00027 | 0.45 |
|  | 5 | 0.71425 | 0.00021 | 0.00028 | 0.45 |
|  | 6 | 0.71456 | 0.00021 | 0.00029 | 0.45 |
|  | 7 | 0.71462 | 0.00020 | 0.00027 | 0.49 |
|  | 8 | 0.71492 | 0.00018 | 0.00025 | 0.49 |
|  | 9 | 0.71516 | 0.00019 | 0.00026 | 0.48 |
|  | 10 | 0.71551 | 0.00020 | 0.00027 | 0.49 |
|  | 11 | 0.71517 | 0.00018 | 0.00025 | 0.49 |
|  | 12 | 0.71497 | 0.00019 | 0.00025 | 0.45 |
|  | 13 | 0.71467 | 0.00020 | 0.00027 | 0.41 |
|  | 14 | 0.71514 | 0.00023 | 0.00032 | 0.40 |
|  | 15 | 0.71535 | 0.00021 | 0.00029 | 0.40 |
|  | 16 | 0.71573 | 0.00021 | 0.00028 | 0.40 |
|  | 17 | 0.71614 | 0.00020 | 0.00027 | 0.42 |
|  | 18 | 0.71617 | 0.00022 | 0.00030 | 0.39 |
|  | 19 | 0.71553 | 0.00024 | 0.00032 | 0.37 |
|  | 20 | 0.71461 | 0.00024 | 0.00032 | 0.46 |
| *dentine* | *21* | *0.71202* | *0.00012* | *0.00016* | *1.14* |

Supplementary Table 3 Space in µm between the sampling lines and sampling time for each sample.

| **Sample** | **Space between lines** | **Sampling time** |
| --- | --- | --- |
| Seal 015 | 200 µm lines 1-24 and 25-26, 600 µm lines 24-25 | ~125sec/line |
| Seal 009 | 600 µm lines 1-4, 200 µm lines 4-29 | ~125sec/line |
| Seal 004 | 200 µm lines 1-14 and 15-40, 600 µm lines 14-15 | 90 - 140 sec/line |
| Seal 029 | 700 µm lines 1-3, 200 µm lines 4-25 | 130 sec/line |
| Seal 013 | 700 µm lines 1-3, 200 µm lines 4-14 | 130 sec/line |
| Seal 006 | 600 µm lines 1-2, 200 µm lines 3-21 | 110 - 140 sec/line |
